# Left-sided portal hypertension caused by peripancreatic lymph node tuberculosis misdiagnosed as pancreatic cancer: a case report and literature review

**DOI:** 10.1186/s12876-020-01420-x

**Published:** 2020-08-18

**Authors:** Dajun Yu, Xiaolan Li, Jianping Gong, Jinzheng Li, Fei Xie, Jiejun Hu

**Affiliations:** 1 Department of General Surgery, Wushan County People’s Hospital of Chongqing, Wushan, Chongqing, 404700 China; 2 Department of Hepatobiliary Surgery, The Second Affiliated Hospital of Chongqing Medical University, 74 Linjiang Road, Yuzhong District, Chongqing, 400010 China; 3 Department of Hepatobiliary Surgery, The First People’s Hospital of Neijiang, Neijiang, 64100 Sichuan China

**Keywords:** Case report, Left-sided portal hypertension, Lymph node tuberculosis, Pancreatic cancer

## Abstract

**Background:**

Left-sided portal hypertension (LSPH) is an extremely rare clinical syndrome, and it is the only form of curable portal hypertension. It is primarily caused by pancreatic disease, and is associated with complications that cause spleen vein compression. Specific symptoms are often lacking, rendering it difficult to diagnose. Splenectomy is the main treatment for cases complicated by variceal bleeding, and the effects of treatment primarily depend on the condition of the primary disease.

**Case presentation:**

The patient was a 29-year-old woman who was admitted to the hospital for repeated hematemesis and black stool. She had been misdiagnosed with pancreatic cancer 7 years prior. Combined imaging and endoscopic examination indicated varicose gastric fundus veins, a pancreatic mass, and enlarged peripancreatic lymph nodes. Laboratory investigations revealed reduced erythrocyte, platelet, and leukocyte counts, the interferon gamma release assay was positive, and liver function was normal. Abdominal exploration, splenectomy, varicose vein dissection, and lesion resection were performed via laparotomy. Postoperative biopsy analysis confirmed the diagnosis of lymph node tuberculosis. Based on the above-described factors, LSPH caused by peripancreatic lymph node tuberculosis was a diagnosed.

**Conclusions:**

Herein we describe the first reported case of LSPH caused by peripancreatic lymph node tuberculosis. When left portal hypertension occurs simultaneously, peripancreatic lymph node tuberculosis is often misdiagnosed as pancreatic cancer. Further studies are necessary to develop a more favorable diagnostic method for pancreas masses and more advantageous therapy for LSPH, especially in cases caused by mechanical compression.

## Background

Lymph nodes are the sites most frequently affected by *Mycobacterium tuberculosis* outside the lung [1–4], but intra-abdominal lymph node tuberculosis is an extremely rare disease and most cases are associated with immunosuppression [5]. Left-sided portal hypertension (LSPH) is a rare clinical syndrome that can lead to bleeding from isolated gastric varices with normal liver function [6]. The most common causes of LSPH include chronic pancreatitis, pancreatic pseudocysts, and various pancreatic tumors [7–12].

Most patients with LSPH are asymptomatic, and only a few patients exhibit isolated gastric varices, ruptures, and fatal bleeding caused by splenic vein obstruction resulting from thrombosis, mechanical compression, tumor invasion, and metastasis [7–14]. It is difficult to diagnose LSPH [15], and bleeding from LSPH is frequently fatal [8, 13, 16–19]. Diseases resulting in LSPH often need to be distinguished from pancreatic cancer, therefore it is necessary for due attention to be paid to the diagnosis and treatment of LSPH [20–22]. Herein we describe a case of LSPH caused by peripancreatic lymph node tuberculosis that had been misdiagnosed as pancreatic cancer 7 years prior.

## Case presentation

The patient was a 29-year-old woman who had undergone debridement and drainage for cervical lymph node tuberculosis 9 years prior to the current presentation. She had been examined 7 years prior to the current presentation via abdominal computed tomography (CT) at another hospital due to abdominal pain. That CT examination revealed a mass in the pancreas body and enlarged lymph nodes in the abdominal cavity. Understandably, at that time it was suspected that she had pancreatic cancer with lymphatic metastasis. Apart from the CT report however, the specific clinical examination index of the patient compiled at the other hospital was unavailable. Two years after the symptoms had been relieved via treatment with traditional Chinese medicine, the patient began experiencing repeated vomiting and melena that had persisted for the subsequent 5 five years; up to the time of the current presentation. At our hospital she stated that her psychological status, appetite, and sleep were normal, and she exhibited ochrodermia but no fever, jaundice, petechiae, or ecchymoses. On physical examination her general condition was good, and there was no abdominal tenderness, abdominal muscle tension, rebound pain, abdominal mass, or hepatomegaly. There was also no swelling of the cervical, supraclavicular, axillary, or inguinal lymph nodes.

To clarify the cause of the patient’s condition, biochemical blood analysis and routine blood examinations were performed. Adenosine deaminase and liver function were within normal ranges (Table 1), but erythrocyte, platelet, and leukocyte counts were reduced (Table 2). Alpha fetal protein, tumor associated antigen 125, and tumor associated antigen 199 results were normal. Tests for human immunodeficiency virus, hepatitis B virus, hepatitis C virus, and *M. tuberculosis* infection were negative, but an interferon gamma release assay was positive.

**Table 1:** Blood biochemical results

Variable	Result	Units	Reference
Total bilirubin	9.4	umol/L	0.0–22.3
Direct bilirubin	7.8	umol/L	0.0–8.6
Indirect bilirubin	1.6	umol/L	0.0–17.1
Serum total bile acid	10.5	umol/L	0.0–10.0
Alanine aminotransferase	21.0	U/L	5.0–40.0
Aspartate aminotransferase	25.0	U/L	5.0–40.0
Alkaline phosphatase	50	U/L	45–125
Glutamyl transpeptidase	11	U/L	5–60
Lactate dehydrogenase	209	U/L	100–350
Cholinesterase	4426	U/L	3930–1380
Adenosine deaminase	10.0	U/L	4.0–22.0
Alpha-l-fucosidase	26.0	U/L	0.0–40.0
Monoamine oxidase	3.0	U/L	0.0–12.0
Prealbumin	181	mg/L	150–380
Total protein	65.8	g/L	65.0–85.0
Albumin	44.4	g/L	40.0–55.0
Globin	21.4	g/L	20.0–40.0
Albumin/Globin	2.07		1.20–2.40

Liver function and other blood biochemical indexes were normal

**Table 2:** Routine blood examination

Variable	Results	Units	Reference
White blood cells	1.81	^*^10 ~ 9/L	3.50–9.50
Red blood cells	2.60	^*^10 ~ 12/L	3.80–5.10
Hemoglobin	67	g/L	115–150
Platelets	50.00	^*^10 ~ 9/L	85.00–350.00
Hematocrit	23.5	%	35.0–45.0
Mean corpuscular volume	90.40	fL	82.00–100.00
Mean corpuscular hemoglobin	25.80	pg	27.00–34.00
Mean corpuscular-hemoglobin concentration	285.000	g/L	316.00–354.00
Red cell volume distribution width-CV	17.90		0.00–15.00
Red cell volume distribution width-SD	59.10		0.00–45.00
Neutrophil ratio	77.90	%	40.00–75.00
Lymphocyte ratio	15.50	%	20.00–50.00
Monocyte ratio	5.50	%	3.00–10.00
Eosinophil ratio	1.10	%	0.40–8.00
Basophil ratio	0.00	%	0.00–1.00
Neutrophils	1.41	^*^10 ~ 9/L	1.80–6.30
Lymphocytes	0.28	^*^10 ~ 9/L	1.10–3.20
Monocytes	0.10	^*^10 ~ 9/L	0.10–0.60
Eosinophils	0.02	^*^10 ~ 9/L	0.02–0.52
Basophil	0.00	^*^10 ~ 9/L	0.00–0.06

White blood cells, red blood cells, hemoglobin, and platelets were reduced, as well as red cell volume distribution width widening

**Fig. 1:**
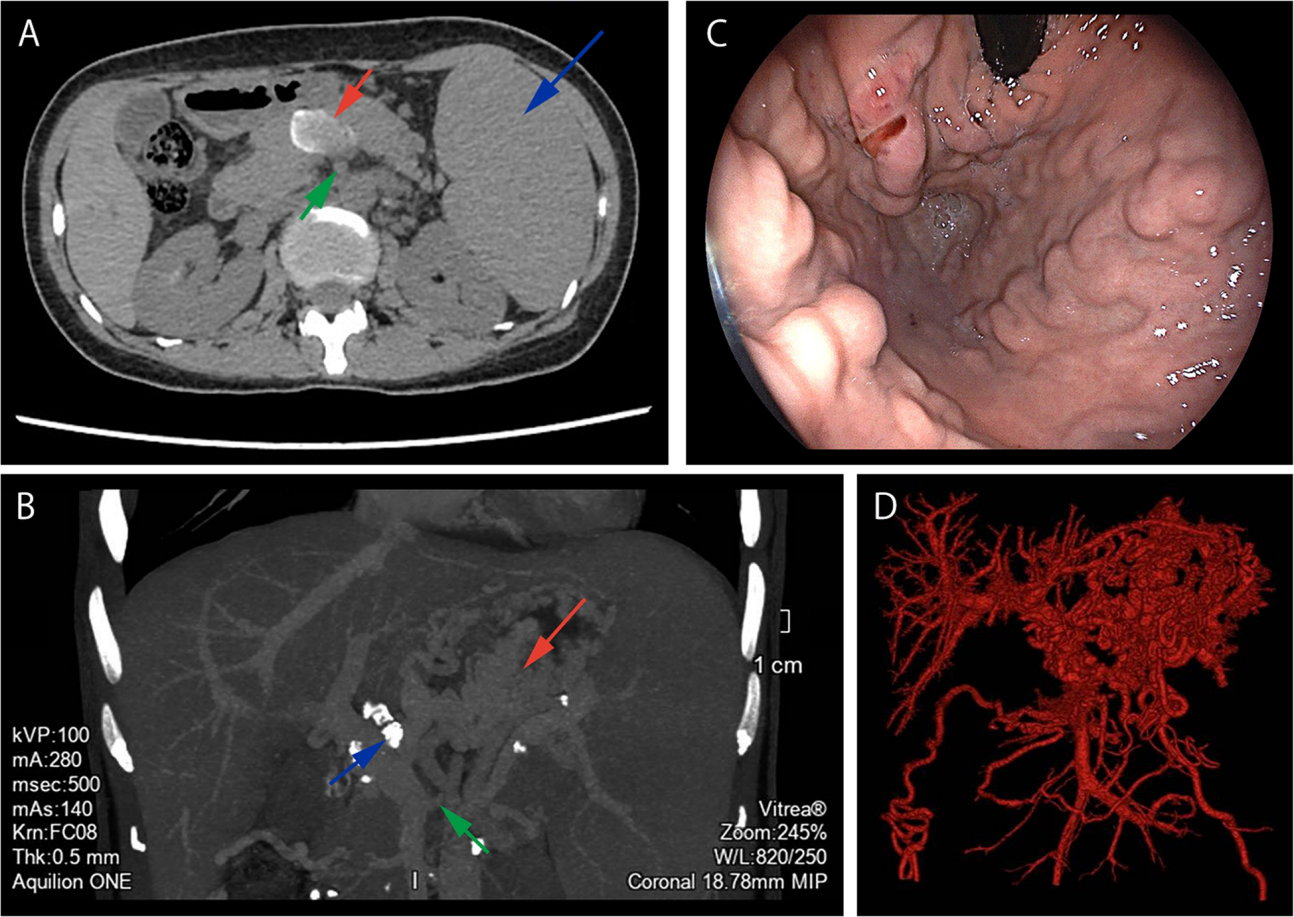
Computed tomography and gastroscopy images. **a** Non-contrast computed tomography imaging on a horizontal plane. There is a low-density and heterogeneous mass in the pancreas body with an irregular margin (red arrow). The spleen is enlarged (blue arrow), and there are dense nodular shadows around the pancreas (green arrow). **b** Computed tomography angiography image in the portal venous phase obtained in a coronal plane. Varicose gastric veins (red arrow) and swollen calcified lymph nodes (blue arrow) are evident. The initial section of the splenic vein becomes narrow (green arrow). **c** Gastroscopy image depicting a varicose vein in the gastric fundus. **d** Portal-phase three-dimensional vascular reconstruction image depicting varicose gastric veins and normal esophageal portal veins

To further aid in the diagnosis, imaging examinations including abdominal non-contrast and contrast-enhanced CT, CT angiography (CTA), CT venography, and portal-phase three-dimensional vascular reconstruction were performed. Both non-contrast and contrast-enhanced CT indicated non-obvious enhancement in both the arterial and venous phases, and a heterogeneous, non-vascular, low-density mass was depicted in the lower margin of the pancreas body with poorly defined edges and dimensions of approximately 3.1 × 2.0 cm (Fig. 1a). An intumescent spleen and multiple nodular dense shadows around the pancreas, hepatic hilar region, and mesentery were also visible on CT (Fig. 1a). CTA, CT venography, and portal-phase three-dimensional vascular reconstruction depicted an enlarged splenic vein, narrowed initial section of the splenic vein, and tortuous gastric veins (Fig. 1b and d). Gastroscopy detected varicose veins under the gastric fundus mucosa (Fig. 1c), but the esophageal mucosa was smooth. Chest CT was conducted to investigate a potential history of tuberculosis, and it depicted a cable-like increased density of flaky shadows in the posterior segment of the upper lobe tip of the left lung. Based on the above results the patient was diagnosed with LSPH. Due to the nature of the mass in the pancreas however, it was unclear whether it was tuberculosis, a tumor, or another lesion. In the present case the mass was located in the lower margin of the pancreas body, and peripancreatic blood vessels were abundant, so it is difficult and dangerous to operate aspiration. According to our Multiple Disciplinary Team, endoscopic ultrasound-guided fine-needle aspiration biopsy (EUS-FNAB) was not applicative.

**Fig. 2:**
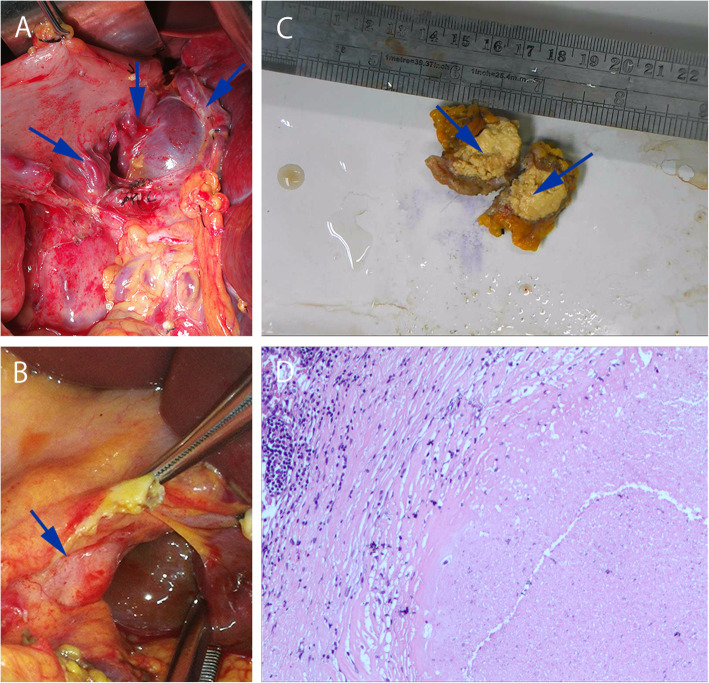
Intraoperative observations and specimens. **a** Varicose gastric veins (blue arrow). **b** An enlarged peripancreatic lymph node (blue arrow). **c** Macroscopic aspects of the swelling in the peripancreatic lymph nodes. The swollen peripancreatic lymph nodes exhibit caseous necrosis (blue arrow). **d** Hematoxylin-eosin staining reveals red staining and structureless particles in the background of lymphocytes

Due to repeated hematemesis symptoms, after a blood transfusion and the improvement of anemia the patient underwent a splenectomy and perigastric fundus vascular dissection, and a lesion excision was performed for biopsy. Intraoperatively an enlarged spleen with dimensions of approximately 34 × 25 × 15 cm was observed. After dissociating the peritoneal adipose tissue it was evident that the left gastric vein, right gastric vein, left gastroepiploic vein, and right gastroepiploic vein were extensively tortuous and dilated (Fig. 2a). As the dissection deepened, swelling of the lymph nodes at the greater curvature, hepatoduodenal ligament, and lower margin of the pancreas were apparent (Fig. 2b). Intraoperative ultrasound suggested that the mass occupying the lower margin of the pancreas was an abscess. Because it exhibited a caseous necrosis profile the sample was submitted for biopsy, and a necrotizing granulomatous lymphadenitis compatible with tuberculosis was observed (Fig. 2c and d). After obtaining these results microbiological analysis was performed, and Ziehl–Neelsen staining was suspiciously positive. Therefore, the diagnosis of LSPH caused by lymph node tuberculosis was confirmed. Based on the newly generated clinical evidence it was concluded that the patient’s diagnosis 7 years prior should have been peripancreatic lymph node tuberculosis rather than pancreatic cancer.

## Discussion and conclusions

Tuberculosis is a common disease in undeveloped countries. Due to increasing immigration and cases of HIV-mediated immunosuppression its incidence has recently increased in western countries [23], thus a concurrent increase in the misdiagnosis of tuberculosis can be expected in these countries. Pulmonary tuberculosis is the most frequent presenting form, and intra-abdominal forms of tuberculosis are uncommon [24–26]. Intra-abdominal tuberculosis occurs in 3.5% of patients with extra-pulmonary tuberculosis [27], and patients with the condition can present with a myriad of non-specific symptoms so diagnosis requires a high level of suspicion [28].

Because of the above-described considerations intra-abdominal tuberculosis has evidently often been misdiagnosed as a tumor such as intra-abdominal lymphoma, gastrointestinal stromal tumor, ovarian carcinoma, and particularly, peritoneal carcinomatosis [29–33]. Intra-abdominal tuberculosis frequently causes nonspecific symptoms, and these symptoms mainly result from mechanical pull and compression. Konstantara et al. [34] reported a case of small intestine volvulus due to intra-abdominal lymphatic tuberculosis, and obstruction jaundice caused by intra-abdominal tuberculosis has also been reported [35]. The current patient who suffered from LSPH resulting from peripancreatic lymph node tuberculosis had been misdiagnosed with pancreatic cancer 7 years prior at another hospital.

Due to a lack of specific symptoms, patients with peripancreatic lymph node tuberculosis primarily present with abdominal pain, constitutional syndrome, jaundice, emaciation, and pancreatitis or an abdominal mass, which are similar to symptoms of pancreatic cancer. There is also a high degree of similarity between the radiological manifestations of peripancreatic lymph node tuberculosis and pancreatic cancer [20, 25, 36–39]. Due to all of these factors peripancreatic lymph node tuberculosis is frequently misdiagnosed as pancreatic cancer. Notably however, the therapeutic approaches to these two diseases are completely different; thus it is important to make a correct diagnosis, in order to avoid unnecessary surgery and long-term complications. For these reasons EUS-FNAB was utilized to distinguish peripancreatic lymph node tuberculosis from pancreatic cancer via Ziehl–Neelsen staining and *M. tuberculosis* culture [39–44].

EUS-FNAB has higher specificity and sensitivity for the differential diagnosis of pancreatic mass than traditional imaging techniques, and it has now been utilized extensively [45–48]. It was the use of EUS-FNAB that prevented the application of unnecessary surgery. Notably however, the accuracy of these tests depends on sample quality and *M. tuberculosis* activity, and the application of EUS-FNAB for aspiration can be difficult at some sites [49]. In the present case the mass was located in the lower margin of the pancreas body, and peripancreatic blood vessels were abundant, so it is difficult and dangerous to operate aspiration. The surgery was mainly designed to relieve gastrointestinal bleeding caused by LSPH, but it ultimately facilitated a specific diagnosis in the present case. Laparoscopic surgery may be a better option in cases in which surgery is performed solely for diagnostic purposes. Moreover, tuberculosis history, adenosine deaminase, the interferon gamma release assay, and tuberculous polymerase chain reaction analysis can be useful for the diagnosis of lymph node tuberculosis [3, 50].

In patients with normal liver function LSPH usually results from splenic vein obstruction or pancreatic inflammatory or neoplastic disease [6]. In the current patient swollen lymph node compression or fibrous scarring after caseous necrosis were the most likely causes. The diagnosis of LSPH is based on clinical, biochemical, and radiological evaluations. Many patients with LSPH are asymptomatic or have primary disease symptoms [13, 14, 51], but in the few LSPH patients who express isolated gastric venous bleeding and anemia the bleeding is usually substantial [6, 9, 12].

Routine blood tests can reveal reductions in red blood cells, lymphocytes, and platelets. Biochemical evaluation is mainly used to exclude cirrhotic portal hypertension and identify primary disease. In addition to clinical symptoms, imaging plays an important role in confirming the diagnosis in the majority of cases [52]. Although angiography of the splenic vein remains the gold standard for diagnosing LSPH, it is now rarely used because it is invasive and entails a possibility of morbidity [53]. Transabdominal ultrasonography is often the initial imaging modality utilized, but its accuracy for the detection of splenic or superior mesenteric vein thrombosis is questionable [54].

Endoscopic ultrasonography has recently been used to evaluate the portal vasculature, and it is reportedly more accurate than transabdominal ultrasonography for evaluating the patency of the splenic vein [13, 55]. With the rapid development of both CT and endoscopy, the combination of multidetector CTA, gastroscopy, and portal-phase three-dimensional vascular reconstruction may be a better option, as suggested by the current case. CTA and portal-phase three-dimensional vascular reconstruction results can reportedly guide the operation if necessary. Magnetic resonance angiography is a promising method for evaluating the portal venous system [56].

Whether asymptomatic patients require treatment remains controversial, but it is necessary to intervene to prevent active bleeding. In addition to addressing bleeding, it is often necessary to treat the primary disease [16, 17, 19, 57, 58]. Based on the patient’s clinical condition, there are several methods that can be used to relieve isolated gastric bleeding. To reduce venous blood reflux, splenectomy remains the preferred treatment for patients with gastric bleeding due to LSPH, and splenic artery embolization can be used as a supplementary measure in patients in whom splenectomy is deemed unsuitable [13, 16, 17, 19, 57–59]. Moreover, while endoscopic therapy is highly advantageous for the treatment of acute massive gastric bleeding, rebleeding is unavoidable [60–63]. We are also currently investigating whether LSPH due to mechanical compression can be corrected via stent implantation. In the therapeutic procedures utilized in the current patient, a splenectomy and varicose vein dissection were performed for hypersplenism and severe varices intervention, but notably the treatment of each patient with LSPH should be individually tailored to maximize the benefits conferred to that individual patient.

In conclusion, herein we have described a very interesting case. Due to a prior misdiagnosis a 29-year-old woman suffered from LSPH, which resulted from peripancreatic lymph node tuberculosis. LSPH is an extremely rare clinical syndrome, and the current case constitutes the first reported account of LSPH caused by peripancreatic lymph node tuberculosis. The dissemination of the details of this case is necessary in order to provide a reference for the clinical diagnosis and treatment of LSPH.

## Data Availability

Not applicable.

## Abbreviations

CT: Computed tomography
CTA: Computed tomography angiography
EUS-FNAB: Endoscopic ultrasound-guided fine-needle aspiration biopsy
LSPH: Left-sided portal hypertension
